# Prevalence of intestinal parasitic infections and risk factors among schoolchildren at the University of Gondar Community School, Northwest Ethiopia: a cross-sectional study

**DOI:** 10.1186/1471-2458-13-304

**Published:** 2013-04-05

**Authors:** Aschalew Gelaw, Belay Anagaw, Bethel Nigussie, Betrearon Silesh, Atnad Yirga, Meseret Alem, Mengistu Endris, Baye Gelaw

**Affiliations:** 1 Department of Medical Microbiology, School of Biomedical and Laboratory Sciences, University of Gondar, Gondar, Ethiopia; 2 Department of Clinical Chemistry, School of Biomedical and Laboratory Sciences, University of Gondar, Gondar, Ethiopia; 3 Department of Parasitology, School of Biomedical and Laboratory Sciences, University of Gondar, Gondar, Ethiopia; 4 Department of Hematology, School of Biomedical and Laboratory Sciences, University of Gondar, Gondar, Ethiopia

**Keywords:** Intestinal parasites, Schoolchildren, Associated risk factors, Gondar, Ethiopia

## Abstract

**Background:**

Intestinal parasitic infections are among the major public health problems in Sub-Saharan Africa. Their distribution is mainly associated with poor personal hygiene, environmental sanitation and limited access to clean water. Indeed, epidemiological information on the prevalence of various intestinal parasitic infections in different localities is a prerequisite to develop appropriate control measures. Therefore, the aim of this study was to assess the prevalence of intestinal parasitic infections and associated risk factors among schoolchildren.

**Method:**

This school-based cross-sectional study was undertaken at the University of Gondar Community School from April 2012 to June 2012. Study subjects were selected using a systematic random sampling method. Data were gathered through direct interview by using a pretested questionnaire. The collected stool specimens were examined microscopically for the presence of eggs, cysts and trophozoites of intestinal parasites using direct saline smear and formol-ether concentration methods. Data entry and analysis were done using SPSS version 16 software.

**Result:**

Out of 304 study subjects, 104 (34.2%) were infected with one or more intestinal parasites. The prevalence rate was 43 (32.1%) for male and 61 (35.9%) for female. The prevalence of intestinal parasites was high in age group of 10–12 years compared to other age groups. The predominant intestinal parasite was *Hymenolepis nana,* followed by *Entamoeba histolytica/dispar* and *Ascaris lumbricoides* with 42 (13.8%), 28 (9.2%), 18 (5.9%), respectively. Hand washing practice and ways of transportation were statistically associated with intestinal parasitic infections. Children in grades 1 to 3 had a higher prevalence of intestinal helminthic infection than those in grades 4 to 8 (p = 0.031).

**Conclusions:**

Intestinal parasites were prevalent in varying magnitude among the schoolchildren. The prevalence of infections were higher for helminths compared to protozoa. Measures including education on personal hygiene, environmental sanitation, water supply and treatment should be taken into account to reduce the prevalence of intestinal parasites.

## Background

Globally two billion individuals were infected with intestinal parasites; out of these majorities were children in resource-poor settings [1]. Particularly in Sub-Saharan Africa, parasitic infections are the major public health problem. Apart from causing morbidity and mortality, infections with intestinal parasites have been associated with stunting, physical weakness and low educational performance of schoolchildren [2,3]. Furthermore, chronic intestinal parasitic infections have become the subject of speculation and investigation in relation to the spreading and severity of other infectious diseases such as HIV/AIDS and leprosy [4,5].

Parasitic infections are governed by behavioral, biological, environmental, socioeconomic and health systems factors. Local conditions such as quality of domestic and village infrastructure; economic factors such as monthly income, employment and occupation and social factors such as education influence the risk of infection, disease transmission and associated morbidity and mortality [6,7]. These infections are more prevalent among the poor segments of the population. They are closely associated with low household income, poor personal and environmental sanitation, and overcrowding, limited access to clean water, tropical climate and low altitude. Intestinal parasitic infections such as amoebiasis, ascariasis, hookworm infection and trichiuriasis are among the ten most common infections in the world [8].

Prevalence of intestinal helminths and other intestinal parasites have been studied in different areas of the tropics and subtropics [9]. Decades ago, a number of studies focusing on intestinal parasites were done in different community groups such as preschool children, schoolchildren, camps and refugees in Northwest Ethiopia [9-11]. In line with this, interventions were undertaken to minimize the burden. Although it is not regularly implemented, there is a deworming program in schools and villages in the study area. However, assessing the effects of interventions taken and trends in the prevalence of intestinal parasites among schoolchildren in Northwest Ethiopia seems sidelined. Therefore, the aim of this study was to determine the prevalence and risk factors of intestinal parasites among schoolchildren. The findings of this study might help in strengthening the information available so far and encourage policy makers to design effective strategies to combat intestinal parasitic infections in the study area.

## Methods

### Study area, design and period

A cross-sectional study was conducted at the University of Gondar Community School in Gondar town from April 2012 to June 2012. The town is located 730 km from Addis Ababa to the Northwest. Gondar is situated at an altitude of 2,185m above sea level. It has a total population of 138,000 and the majority of the population is Amhara by ethnicity. This community school is found 6 km from the center of Gondar town to the Southwest. The school offers education from kindergarten to high school level. There were seven hundred thirty students enrolled during the study period. These students are from families with different economic backgrounds.

### Sample size and sampling technique

The sample size (n) was estimated using the single population proportion formula Z^2^ p (1-p) / d^2^. Where P = prevalence of intestinal parasites from previous study, d = margin of error and Z = standard score corresponds to 1.96. This gave a sample size of 310. The prevalence rate (p) of intestinal parasites from previous study in the area was 72% [10]. For the calculation, a 95% confidence interval and a 5% margin of error were used. To minimize errors arising from the likelihood of non-compliance, five percent of the sample size was added giving a final sample size of 326.

To select the children, the students were stratified according to their educational level (grade 1 to grade 8). A quota was then allocated for each grade with proportional allocation according to the number of students in each grade. Finally, the participating children were selected using systematic random sampling technique by using class rosters as a sample frame.

### Data collection and laboratory processing

Based on the possible risk factors the questionnaire was developed. This questionnaire was tested for validity by interviewing ten students from Mesert Elementary School. Two senior laboratory technologists who were trained for this purpose collected the data. To ensure the reliability of the information, the children were interviewed in their mother tongue. The interview included information such as age, sex, religion, hands washing habit, ways of transportation, family monthly income and swimming habit. At the time of conversation, interviewers also inspected whether the fingernails of the students were trimmed and their feet were covered with shoes. All the questionnaires were checked for accuracy and completeness.

After proper instruction, the children were given labelled collection cups and applicator sticks. From each student, about 2g of fresh stool was collected. Each of the specimens was checked for its label, quantity and procedure of collection. A portion each of the stool samples was processed with a direct microscopic technique to detect cysts, trophozoites, eggs and larva of intestinal parasites immediately. The remaining part of the samples were preserved in 10% formalin solution and transported to the University of Gondar Research Laboratory. Stool examinations were performed using the formol-ether concentration technique [12]. Two trained senior medical laboratory technologists examined the samples microscopically. Both the 10× and 40× objectives were used for detection of eggs and larvae of helminth and cysts and trophozoites of protozoan parasites. Iodine solution was used to detect and identify cysts of protozoan parasites.

### Data management and analysis

The data were entered and analyzed using the SPSS statistical software version 16. In all cases, *P*-values less than 0.05 were considered statistically significant. Initially the association between each exposure and the presence of infection was assessed using the Chi-squared test. Odds ratios were computed to measure the strength of association. To determine independent risk factors for infection, logistic regression analysis was employed.

### Ethical considerations

This study was approved by the ethical review committee of the School of Biomedical and Laboratory Sciences, University of Gondar. Official letter was submitted to the University of Gondar community school. Informed verbal consent was obtained from parents/guardians of the students. The purpose and the importance of the study were explained and permission was obtained from the Director of the school. A medical doctor from the University of Gondar treated those schoolchildren who were positive for intestinal parasites based on the national protocol.

## Results

### Socio demographic characteristics

A total of 326 students were selected for investigation. Of these 22 (6.75%) were excluded because of production of insufficient specimens. For this reason, only 304 students were ultimately included in the study. Of these, 170 (55.9%) were males and the rest 134 (44.1%) were females. The mean age of the study participants was 9.5 years (Table 1).

**Table 1:** Socio-demographic characteristics of children at the University of Gondar Community School, Gondar town, Northwest Ethiopia, April- June 2012

**Socio-demographic characteristics**	**Number of students**	**Percentage**
Gender		
Male	170	55.9
Female	134	44.1
Age group		
≤9	132	43..4
10-12	118	38.8
≥13	54	17.8
Religion		
Christian	272	89.5
Muslim	32	10.5
Level of education (Grade)		
1	37	12.2
2	38	12.5
3	40	13.2
4	38	12.5
5	36	11.7
6	38	12,5
7	37	12.2
8	40	13.2

### Prevalence of intestinal parasites

Various types of intestinal parasites including protozoans, trematodes, cestodes and nematodes were detected. The prevalence of *Hymenolepis nana* infection was 42 (13.8%) followed by 28 (9.2%) of *Entamoeba histolytica/dispar. Hymenolepis nana*, *Entamoeba histolytica/dispar* and *Ascaris lumbricoides* were detected as single infections in 35 (11.5%), 22 (7.2%), and 12 (3.9%) individuals, respectively. For both sexes, the proportion of infections was higher for helminths compared to protozoa, i.e. about 1.64 times (Table 2 and 3). Overall, one-third, (104 (34.2%)) of the students were infected with intestinal parasites and 88 (28.9%), 8 (2.6%) and 6 (2.0%) of the students had single, double and triple infections, respectively. *Trichuris trichiura* with *Hymenolepis nana*, *Ascaris lumbricoides* with *Hymenolepis nana*, *Ascaris lumbricoides* with *hookworm* and *Giardia intestinalis* with *Entamoeba histolytica/dispar* comprised 4 (1.3%), 3 (1.0%), 3 (1.0%) and 4 (1.3%) of the double infections, respectively. *Ascaris lumbricoides* and *Trichuris trichiura* were found with *Hymenolepis nana* and *Entamoeba histolytica/dispar* as triple infections. In all age groups, the predominant intestinal parasite detected was *Entamoeba histolytica/dispar*, followed by *Hymenolepis nana* and *Ascaris lumbricoides* (Table 2).

**Table 2:** Distribution of intestinal parasites among schoolchildren at the Gondar University Community School, Northwest Ethiopia, April- June 2012

**Types of parasite**	**Male**	**(n = 170)**	**Female**	**(n = 134)**	**Total (n = 304) No(%)**
	**No**	**%**	**No**	**%**	
**Protozoa**	**20**	**11.8**	**20**	**14.9**	**40 (13.2)**
*E. histolytica/dispar*	12	7.1	16	12.0	28 (9.2)
*G. intestinalis*	8	4.7	4	3.0	12 (3.9)
**Helminthes**	**54**	**31.8**	**28**	**20.9**	**82 (26.9)**
*A.lumbricoides*	10	5.9	8	6.0	18 ( 5.9)
*T. trichiura*	6	8.6	4	3.0	10 (3.2)
*S. stercoralis*	2	1.2	0	0	2 (0.7)
*Hookworm*	4	2.4	2	1.4	6 (2)
*H. nana*	28	16.5	14	10.4	42 (13.8)
*S. mansoni*	4	2.4	0	0	4 (1.3)
**Total**	**61**	**35.9**	**43**	**32.1**	**104 (34.2)**

NB: The sum of the columns is greater than the total because of the coinfection of some of the students.

**Table 3:** Distribution of intestinal parasites by age among children at the University of Gondar Community School, Gondar town, Northwest Ethiopia, April - June 2012

**Type of parasite**	**Age group in years**			**Total ( n = 304) No(%)**
	**≤ 9 (n = 132)**	**10-12 (n = 118)**	**≥13 (n = 54)**	
	**No(%)**	**No(%)**	**No(%)**	
**Helminthes**	**28 (21.2)**	**34 (24.6)**	**23 (42.6)**	**82 (26.9)**
*H. nana*	18 (13.6)	16 (13.6)	8 (14.8)	42 (13.8)
*A.lumbricoides*	4 (3.0)	10 (8.5)	4 (7.4)	18 (5.9)
*S. mansoni*	0 (0)	2 (1.7)	2 (3.7)	4 (1.3)
*S. stercoralis*	0 (0)	1 (0.8)	1 (1.8)	2 (0.7)
*T. trichiura*	5 (3.8)	3 (2.5)	2 (3.7)	10 (3.3)
*Hookworm*	1 (0.8)	3 (2.5)	2 (3.7)	6 (2.0)
**Protozoans**	**16 (12.1)**	**17 (14.4)**	**9 (16.7)**	**40 (13.2)**
*G. intestinalis*	4 (3.0)	5 (3.9)	3 (3.6)	12 (3.9)
*Entamoeba histolytica/dispar*	12 (9.1)	12 (10.2)	4 (7.4)	28 (9.2)
**Total**	**39 (29.5)**	**50 (42.3)**	**13 (24.1)**	**104 (34.2)**

NB: The sum of the columns is greater than the total because of the coinfection of some of the students.

### Intestinal parasite and possible risk factors

The demographics and risk factors of the study subjects are shown in Table 4. One hundred twenty four (40.8%) of the children reported hand washing with soap and water before meals and after defecation. The odds of intestinal parasitic infection in children who do not practice hand washing before eating is 6.45 times higher than those who practice it (p = 0.0076, 95% CI = 4.55-11.90). Children in grade one to grade three had a higher prevalence of intestinal helminthic infections than those in grades four to eight (p = 0.031). The prevalence of intestinal parasitic infection was not significantly related to, family monthly income and swimming habits (p > 0.05). Further analyses of the data showed that intestinal parasitic infection was independent of family size, religion, or gender of the children (p > 0.05). In the univariate analyses, no statistically significant associations were observed between male versus female (p = 0.301). Although there was no statistically significant association between age and parasitic infection, the age group 10–12 years had a high proportion of parasitic infection compared to other age groups. Variable that appeared to be associated with the outcome variable during bivariate analysis was taken in to multivariate logistic regression for further analysis. In multivariate analysis, none of the observed risk factors was statistically associated with parasitic infection.

**Table 4:** Univariate analysis of intestinal parasitic infections and potential risk factors among children at the University of Gondar Community School, Northwest Ethiopia, April-June 2012

**Risk factor**	**Parasite status**				
	**Positive N = 104**	**Negative N = 200**	**Total N = 304**	**P-Value**	**Crude OR (95% CI )**
Gender					
Female	43 (32.1)	91 (67.9)	134 (44.1)	0.431	1.11 (0.49 - 2.50)
Male	61 (35.9)	109 (64.1)	170 (55.9)		
Age					
≤ 9	41 (31.1)	91 (68.9)	132 (43..4)	0.460	1.91 (0.54 -7.52)
10-12	50 (42.4)	68 (57.6)	118 (38.8)		
≥ 13	13 (24.1)	41 (75.9)	54 (17.8)		
Level of education					
Grade 1-3	74 (64.3)	41 (35.7)	115 (37.3)	0.031	4.20 (3.34 - 8.50)
Grade 4-8	30 (15.9)	159 (84.1)	189 (62.7)		
Number of siblings					
≤ 2	66 (38.4)	106 (61.6)	172 (56.6)	0.081	2.60 (1.12 - 6.06)
≥ 3	38 (28.9)	94 (71.1)	132 (43.4)		
Hand washing habit					
Yes	28 (25.5)	82 (74.5)	110 (36.2)	0.0076	6.45 (4.55- 11.90)
No	76 (39.2)	118 60.8)	194 (63.8)		
Family monthly income (ETB*)					
<1000	46 (38.3)	74 (61.7)	120 (39.5)	0.420	1.59 (0.26-1.36)
1000-2000	39 (32.8)	80 (67.2)	119 (39.1)		
>2000	19 (29.2)	46 (70.8)	65 (21.4)		
Type of transportation					
By car	44 (21.8)	158 (78.2)	202 (66.4)	0.0001	5.3 (2.62- 8.45)
On foot	60 (58.8)	42 (41,2)	102 (33.6)		
Swimming habit					
Yes	21 (32.3)	44 (67.7)	65 (21.4)	0.925	1.03 (0.51- 2.09)
Never swim	83 (34.7)	156 (65.3)	239 (78.6)		
Religion					
Christian	90 (33.3)	182 (67.7)	272 (89.5)	0.795	1.76 (0.85- 3.65)
Muslim	14 (43.8)	18 (56.2)	32 (10.5)		

* Ethiopian Birr.

## Discussion

Epidemiological studies on the prevalence with infection of intestinal parasites in different localities have as a primary objective to identify high-risk communities and formulate appropriate interventions. In line with this view, the present study attempted to assess the prevalence of different intestinal parasitic infections in schoolchildren in a private community school in Gondar town, Northwest Ethiopia, The results of the study showed the occurrence of several intestinal parasites of public health importance among schoolchildren.

The observed prevalence of intestinal parasites of 104 (34.2%) was lower compared with reports of other similar studies, 72.9% in Gondar, Azezo [10], 83% in Jimma [11] and 83.8% in South East of Lake Langano [13]. On the other hand, the prevalence observed in this study was higher than a study conducted in Babile (27.2%) [14]. These variations in prevalence might be due to differences in climatic conditions, environmental sanitation, economic and educational status of parents and study subjects, and previous control efforts.

According to the present study, the prevalence of *Hymenolepis nana, Entamoeba histolytica/dispar,* and *Ascaris lumbricoides* were 13.8%, 9.2%, and 5.9%, respectively. Infections with *Entamoeba histolytica/dispar* was more prevalent among the female population, while *Hymenolepis nana* affected more males but the association was not statistically significant (p > 0.05). These three parasites are prevalent among lower age groups. This finding is in line with a study conducted in Metema District Hospital [15]. On the other hand, the prevalence of *Ascaris lumbricoides, hookworm* and *Schistosoma mansoni* were lower compared to a school-based study done in Azezo, Gondar [10]. This difference might be due to improved environmental sanitation in the community school as compared to Azezo elementary school, regular wearing of shoes and better family income among the study participants.

In this study, multiple infections (polyparasitism) occurred in 14 individuals or 4.6% of the total examined subjects and 13.5% of those who had intestinal parasites. This prevalence is lower compared with similar studies in Gondar, Azezo [10]. Double infections were seen in six study subjects with intestinal parasites. The prevalence of multiple infections was very low compared to previous studies [13,16]. Sample size, study population and the methods used could attribute to this observed difference in detections of various parasites.

In this study, 36.2% of the study participants practice good hand washing before eating and after toilet using soap and water. Intestinal parasitic infections were significantly associated with poor hand washing practice (p < 0.05). The likelihood of acquiring infections among students who do not practice hand washing was 6.45 (95% CI = 4.55- 11.90) times higher than among those who had good hand washing practice. Similarly, ways of transportation to the school had statistically significant association with parasitic infections. This finding is consistent with a study conducted in Babile town [14]. Similar to a report in Zarima town [17], the level of education of the study participants was significantly associated with intestinal parasitic infections. However, family monthly income, number of siblings, religion and swimming habit did not show statistically significant associations with intestinal parasitic infections (P > 0.05) (Table 4).

In this study, protozoan infections were more prevalent in schoolchildren especially in those under 12 years of age while there was reduction as age increased. The reason could be due to the slow development of immunity in adults to protozoan parasites and better awareness in washing hands and other personal hygiene measures. On the other hand, there was no significant difference in the rate of infection due to helminths in relation to the age of students. This might be due to different evasion mechanisms to immunity by helminths.

The present study was subjected to the following limitations. The study was non-blinded. Due to lack of antigen tests, *Entamoeba histolytica* and *Entamoeba dispar* were not separated. As the collection period was short, potential seasonal fluctuations might have affected the actual prevalence. Modified acid-fast staining technique was not used to detect *Cryptosporidium species.*

## Conclusion

This study showed that intestinal parasites were prevalent in varying magnitude among the schoolchildren. The proportions of infection were higher for helminths compared to protozoa. *Hymenolepis nana* among the helminths and *Entamoeba histolytica/dispar* among the protozoans were predominant. Poor hand washing practices and ways of transportation were statistically associated with intestinal parasitic infections. The findings showed that much work remains to be done to improve the health of the students. Measures including education on personal hygiene and environmental sanitation, water supply and treatment should be taken into account to reduce the prevalence.

## Competing interests

The authors declare that they have no competing interests.

## Authors’ contributions

AG, AY, BS MA, and ME conceived the idea for this study. AG, BA, BN, AY, BS, MA and participated in the design and conduct of the study. AG, BG and BA were responsible for the accuracy of the data. AG, BA and ME drafted the manuscript. BN, AY, BS and MA guarantee the statistical analysis. BA, BN, AY, BS, BG and MA interpreted the findings. All authors read and approved the final manuscript.

## Pre-publication history

The pre-publication history for this paper can be accessed here:

http://www.biomedcentral.com/1471-2458/13/304/prepub
